# Probiotic Characteristics and Safety Assessment of *Lacticaseibacillus casei* KGC1201 Isolated from *Panax ginseng*

**DOI:** 10.4014/jmb.2211.11029

**Published:** 2023-01-31

**Authors:** Yun-Seok Lee, Hye-Young Yu, Mijin Kwon, Seung-Ho Lee, Ji-In Park, Jiho Seo, Sang-Kyu Kim

**Affiliations:** 1 Laboratory of Products, Korea Ginseng Corporation, Daejeon 34128, Republic of Korea; 2 Laboratory of Efficacy Research, Korea Ginseng Corporation, Daejeon 34128, Republic of Korea; 3 Laboratory of Analysis, Korea Ginseng Corporation, Daejeon 34128, Republic of Korea; 4 Science Instrumentation Assessment and Application Team, Korea Basic Science Institute (KBSI), Daejeon 34133, Republic of Korea

**Keywords:** Acid-resistance, exopolysaccharide, *Lacticaseibacillus casei*, ginseng, probiotics

## Abstract

*Panax ginseng* is one of the most important herbal medicinal plants consumed as health functional food and can be fermented to achieve better efficacy. *Lacticaseibacillus*, one of the representative genera among lactic acid bacteria (LAB), has also been used as a probiotic material for health functional foods due to its beneficial effects on the human body. To achieve a synergistic effect by using these excellent dietary supplement ingredients together, a novel LAB strain was isolated from the root of 6-year-old ginseng. Through similarity analysis of 16S rRNAs and whole-genome sequences, the strain was confirmed as belonging to the genus *Lacticaseibacillus* and was named *L. casei* KGC1201. KGC1201 not only met all safety standards as food, but also showed excellent probiotic properties such as acid resistance, bile salt resistance, and intestinal adhesion. In particular, KGC1201 exhibited superior acid resistance through morphological observation identifying that the cell surface damage of KGC1201 was less than that of the *L. casei* type strain KCTC3109. Gene expression studies were conducted to elucidate the molecular mechanisms of KGC1201’s acid resistance, and the expression of the glycosyltransferase gene was found to be significantly elevated under acidic conditions. Exopolysaccharides (EPSs) biosynthesized by glycosyltransferase were also increased in KGC1201 compared to KCTC3109, which may contribute to better protection of KGC1201 cells from strong acidity. Therefore, KGC1201, with its increased acid resistance through molecular mechanisms and excellent probiotic properties, can be used in health functional foods to provide greater benefit to overall human health and well-being.

## Introduction

Probiotics are defined as live microorganisms that provide health benefits to the host when ingested in sufficient quantities, according to the Food and Agriculture Organization (FAO) and the World Health Organization (WHO) [1]. Lactic acid bacteria (LAB) are gram-positive, catalase-negative, non-spore-forming, and non-motile organisms [2], and include the genera *Bifidobacterium*, *Carnobacterium*, *Enterococcus*, *Lactobacillus*, *Lactococcus*, *Leuconostoc*, *Oenococcus*, *Pediococcus*, *Streptococcus*, and *Tetragenococcus* [3]. Among these, *Lactobacillus* and *Bifidobacterium* are most commonly used as probiotics [4], which can regulate human immunity [5] and inhibit the growth of pathogens in the gastrointestinal tract and urogenital organs [6]. These microorganisms also participate in intrinsic defense mechanisms by producing hydrogen peroxide, organic acids, diacetyl and bacteriocins [7], and releasing biosurfactants [8].

Effective probiotic microorganisms must be able to pass through the gastrointestinal tract and reach the small or large intestine alive. Gastric acidity reduces the survival rate of microorganisms, including pathogens, but beneficial bacteria that pass through the gastrointestinal tract must withstand acid stress in order to be used as probiotics [9]. Acid-resistance is strain-specific, and organisms can adapt in different ways to promote their survival in acidic environments [10]. The mechanisms associated with acid-resistance in LAB include biofilm formation, proton pump activity, alkaline material production, and neutralization by lactic acid fermentation [11]. Exopolysaccharides (EPSs), which are produced by several members of LAB, are involved in the formation of biofilms and contribute to protection against harsh environments such as the acidic conditions of the stomach [12, 13].

*Panax ginseng* is widely used in Asian countries, including Korea, as a medicinal plant, and its products are popular as health functional food around the world, including North America and Europe [14]. Ginsenosides, the most promising bioactive compounds of ginseng, can be converted into more biologically active forms containing a lesser number of sugar moieties through heating, acid treatment, enzymatic digestion, and microbial fermentation [15]. Nowadays, food-compatible microbes such as LAB are used to convert food-grade deglycosylated ginsenosides [16]. Therefore, using ginseng and LAB together as materials for health functional food can provide a synergistic effect that will help to improve overall health and well-being. Moreover, as *Panax ginseng* has been reported to have high antibacterial activity [17, 18], the isolation of potential probiotic strains derived from ginseng is of growing importance [19, 20].

In this study, the lactic acid bacterium *Lacticaseibacillus casei* KGC1201 (the taxonomic nomenclature of the genus *Lactobacillus* was revised to *Lacticaseibacillus* in April 2020) [21] was newly isolated from the root of *Panax ginseng*. We evaluated the safety and potential of KGC1201 as a probiotic and material for health functional food. We also investigated the genetic and functional characteristics of KGC1201, as well as the molecular mechanisms underlying the superior acid resistance of this strain.

## Materials and Methods

### Isolation and Identification of KGC1201

The strain was isolated from six-year-old ginseng (*Panax ginseng*) roots cultivated at the Korea Ginseng Corporation (KGC) ginseng experimental field (Korea). The collected ginseng roots were disinfected with 70%ethanol and 3% NaClO, and root sections were then fermented for five days at 37°C using purified water supplemented with 1.25% sucrose (w/v) and 2.5% glucose (w/v). The fermentation broth was inoculated into de Man, Rogosa, and Sharpe (MRS) agar medium (BD Difco, USA) with 0.5% CaCO_3_ (w/v), and incubated for 48–72 h at 37°C. For 16S ribosomal RNA (rRNA) gene sequencing of the colonies forming clear zones, the primers 27F (5¢-AGAGTTTGATCCTGGCTCAG-3¢) and 1492R (5¢-GGTTACCTTGTTACGACTT-3¢) were used. Whole-genome sequencing of KGC1201 was performed using an Illumina MiSeq 300 system with 2 × 300-bp paired-end reads using a 600-cycle sequencing kit (MiSeq Reagent Kit v3, CJ Bioscience, Inc., Korea). The assembled genome sequence of KGC1201 was compared with the *L. casei* type strain KCTC3109 genome (accession numbers: NZ_AP012544, NZ_AP012545 and NZ_AP012546) using EzBioCloud average nucleotide identity (ANI) calculator service (CJ Bioscience, https://www.ezbiocloud.net/tools/ani). The strain *L. casei* KGC1201 was deposited with the Korea Collection for Type Culture (KCTC, Korea) with the accession number KCTC14652BP.

### Biochemical Properties

*L. casei* type strain KCTC3109 was acquired from the KCTC (Korea), and used for comparison of biochemical properties with KGC1201. The diluted cell pellets were loaded onto an API 50 CH test strip (bioMérieux, France), and incubated for 48 h at 37°C. The specific carbohydrates fermented by KGC1201 and KCTC3109 were determined by color change on the reagent strip. To investigate the effect of red ginseng extracts (RGEs) on the growth of KGC1201 and KCTC3109, 10 ml MRS broth was prepared containing either 0%, 1% and 2% RGE, and activated cells were inoculated with 1% (v/v) of each medium. The colonies were counted on an MRS agar medium for plate, and RGE effects were presented as relative values proportional to the number of colonies in RGE-free medium.

### Antibiotic Resistance and Virulence Factors

The virulence factors database (VFDB; version 2020.02.13; http://www.mgc.ac.cn/VFs/) was used to identify virulence genes by the BLASTn algorithm with conditions of identity > 70%, coverage > 70%, and E-value < 1E-5 [22]. The ResFinder database (version 4.1; https://cge.food.dtu.dk/services/ResFinder-4.1/) was used to detect antibiotic resistance genes with a threshold of > 90% for %ID and 60% for minimum length [23]. The minimum inhibitory concentration (MIC) test for eight antibiotics, including ampicillin, gentamicin, kanamycin, streptomycin, erythromycin, clindamycin, tetracycline, and chloramphenicol, were performed according to European Food Safety Authority (EFSA) guidelines [24]. Resistance of the strain to each antibiotic was measured using the E-test method with MIC strips (Liofilchem Inc., Italy).

### Safety Assessments

To evaluate the enzymatic profile by APIzym, API ZYM kits (bioMérieux) were used. After culturing, *L. casei* KGC1201 was loaded onto API ZYM strips. Following incubation at 37°C for 3 h, ZYM-A, and ZYM-B reagents were added to each well of plates that were then left at room temperature for 5 min. The color change was observed to determine the corresponding enzyme activity. D-lactate production was evaluated by a D-/L-Lactate (Rapid) Assay Kit (Megazyme Ltd., Ireland). The absorbance of the mixture was measured at 340 nm, and the concentration of D-/L-lactate was calculated according to the manufacturer’s protocol. The hemolytic properties were assessed by the formation of clear zones around the colonies on sheep blood agar plates (KisanBio, Korea). *Escherichia coli* KCTC2441 purchased from the KCTC and *Staphylococcus aureus* NCTC10788 purchased from bioMerieux were used as positive controls of α-hemolysis and β-hemolysis, respectively. Biogenic amines were measured at 254 nm using HPLC (LC-NETI/ADC, UK) with a C18 column (ANPEL Laboratory analysis, Shanghai, China; 4.6 mm × 250 mm C18 column). Acetonitrile solution (67:33 dissolved in water) was used as mobile phase with a flow rate of 0.8 ml/min.

### Bile Salt Resistance and Adhesion Ability to Intestinal Cells

Bile salt resistance was evaluated by comparing the survival rate of bacteria after bile salt exposure, using the following equation: survival rate (%) = [viable cells (log CFU/ml) / initial cells (log CFU/ml)] × 100. The cells were inoculated at 10^8^ CFU/ml in PBS buffer (pH 7.4) with 0.1% (w/v) Oxgall (KisanBio) and cultured in MRS agar medium. The intestinal adhesion properties were evaluated using colonic epithelial cells (HT-29) cultured in RPMI 1640 medium (Hyclone, USA) containing 10% FBS (Hyclone) and 1% penicillin-streptomycin (Thermo Fisher Scientific, USA). The strains attached to the cells were removed using 0.05% trypsin-EDTA solution (w/v), and the number of bacteria was measured as follows: adhesion (%) = [viable cells (log CFU/mL) / initial cells (log CFU/ml)] × 100. All data were obtained from three independent experiments and expressed as mean ± standard error. Significant differences were determined using Student’s *t*-tests and indicated as * *p* < 0.05 and ** *p* < 0.01.

### Acid Resistance

Acid resistance was evaluated by comparing the survival rate of bacteria after acid exposure and using the equation: survival rate (%) = [viable cells (log CFU/ml) / initial cells (log CFU/ml)] × 100. The cells were inoculated at 10^8^ CFU/ml in PBS buffer adjusted to pH 2.0–2.5 using hydrochloric acid (HCl). Morphological characteristics in an acidic environment were analyzed using field emission scanning electron microscopy (FE-SEM; Hitachi S-4800, Japan). The SEM instrument was operated at an accelerating voltage of 3 kV with an emission beam current of 10 μA.

### Quantitative Real-Time Polymerase Chain Reaction (qRT-PCR)

The genome sequence of KCTC3109 was collected from the National Center for Biotechnology Information (NCBI). The primers for the acid resistance genes, such as H^+^/Cl^−^ exchange transporter, histidine kinase, and glycosyltransferase, as well as glyceraldehyde-3-phosphate dehydrogenase (*GAPDH*) as an internal control gene, were designed for qRT-PCR (Table S1). Total RNA was extracted from the cells of KGC1201 and KCTC3109 incubated with control (pH 7.4) or acidic (pH 2.0) PBS buffer for 3 h at 37°C. cDNA was then synthesized and amplified using the 7500 Fast Real-Time PCR System (Applied Biosystems, USA).The relative expressions and fold changes of acid resistance genes were quantified using the comparative C_T_ method [25, 26]. The significances of the differences in relative gene expression levels of two biological and three technical replicates were evaluated using one-way ANOVA and the Tukey HSD test at a 95% confidence interval.

### Purification of EPSs

EPSs were isolated and purified according to the method in previous study with some modifications [27]. The cell pellet and the cell-free supernatant were precipitated twice with 14% trichloroacetic acid (w/v) and 70%ethanol (v/v), respectively, and then dialyzed at 4°C for 1–2 days using an osmotic membrane (Spectra/Por molecular porous tubular dialysis membrane). The dialyzed EPSs derived from the cell pellet and the cell-free supernatant, respectively, were lyophilized at −80°C, and their quantity was measured by weighing.

## Results and Discussion

### Isolation and Identification of LAB from Ginseng

To isolate bacterial strains endogenous to ginseng, six-year-old ginseng roots were fermented using carbohydrates. A total of 243 colonies were isolated, and among them, 195 colonies formed clear zones in CaCO_3_-added MRS medium. Through 16S rRNA sequencing and comparison with the GenBank database, 16 species belonging to 6 genera were obtained, and only one species of the genus *Lacticaseibacillus* was identified (Table S2). Phylogenetic analysis with 16S rRNA sequences of 15 species belonging to *Lacticaseibacillus* showed that the strain was classified into the *Lacticaseibacillus casei* group, and thus it was named *L. casei* KGC1201 (Fig. S1). A comparison of the whole-genome sequences between KGC1201 and *L. casei* type strain KCTC3109 also demonstrated that KGC1201 was highly similar with *L. casei* on a genome-wide scale. The genome size and the GC content ratio of KGC1201 were slightly lower than those of KCTC3109, but the overall genome relatedness between KGC1201 and KCTC3109 determined by ANI was very high at 99.92% (Table S3).

### Biochemical Properties of KGC1201

Carbon source optimization is critical to obtain the highest yield of LAB and its products, such as EPS [28]. To identify the optimal type of carbon source for the growth and metabolism of KGC1201, carbohydrate fermentation tests were performed and biochemical intrinsic properties of KGC1201 were compared with KCTC3109. As a result, three types of carbohydrates—D-maltose, D-melibiose, and D-raffinose—were utilized differently between KGC1201 and KCTC3109 (Table 1). This result may be used to establish the carbon source composition in the culture medium when KGC1201 is used as a health functional food material in the future.

Ginseng and its bioactive compounds, ginsenosides, can also be used as a carbon source for LAB. However, in order to use ginseng and LAB together as materials in health functional food, it is necessary to test not only the ability of LAB to use ginseng as a carbon source, but also the ability to overcome the antibacterial activity of ginseng [17, 18]. Relative growth of KGC1201 increased in proportion to the concentration of red ginseng extracts (RGE), but KCTC3109 had no dependence on the concentration of RGE (Fig. S2). This result indicates that ginseng does not inhibit the growth of KGC1201 isolated from ginseng, but rather promotes its growth by being used as a carbon source.

### Safety Assessment of the Antibiotic Resistance of KGC1201

In rare cases, probiotics can cause side effects in the gastrointestinal tract [29]. It is therefore desirable that organisms used as probiotics do not include genes associated with virulence and antibiotic resistance [30]. In addition, safety evaluation of these genes is necessary to prevent the transfer of drug resistance genes from probiotics to intestinal pathogens [31, 32]. To identify antibiotic resistance genes and virulence factors, the whole genome sequences of KGC1201 were analysed using VFDB and ResFinder database. As a result of in *silico* analysis, there were no antibiotic resistance genes and virulence factors in the genome of KGC1201, suggesting that the safety of KGC1201 has been revealed at the genome level.

Although no antibiotic resistance genes were detected in the genome of KGC1201, MIC tests for 8 antibiotics were additionally performed to confirm that KGC1201 did not actually have antibiotic resistance. As a result, this strain was susceptible to all antibiotics except for kanamycin and streptomycin based on the EFSA cut-off value (Table S4). Since most species belonging to *Lacticaseibacillus* are known to be relatively resistant to aminoglycoside antibiotics such as kanamycin and streptomycin [20, 33, 34], KGC1201 also seems to have inherited this intrinsic characteristic.

### Safety Assessment of Noxious Enzymes, D-Lactate, Hemolysin, and Biogenic Amines

For an organism to be used as a probiotic, safety evaluation for noxious enzymes, D-lactate, hemolysin, and biogenic amines should also be conducted [35]. Noxious enzymes, such as β-glucuronidase, have the capacity to convert procarcinogens to carcinogens through hydrolysis of glycosidic bonds [36]. The accumulation of D-lactate can occur only in case of gastrointestinal dysfunction, such as D-lactate metabolism disorders, but excessive accumulation of D-lactate in the blood can cause health problems [37]. Hemolytic activity, usually caused by hemolysin produced by microorganisms, induces lysis of red blood cells leading to infection by pathogens [38]. Biogenic amines can be rapidly metabolized by the appropriate enzymes in healthy people, but some sensitive individuals can exhibit clinical symptoms even when exposed to them at low doses [39]. KGC1201 did not show any activity against noxious enzymes (Table S5) and mainly produced L-lactate (4.09 mM, 88.9%) rather than D-lactate (0.51 mM, 11.1%). KGC1201 did not show any hemolytic activity on sheep blood agar, whereas *E. coli* and *S. aureus* exhibited α-hemolytic and β-hemolytic activities, respectively (Fig. S3). Biogenic amines such as tyramine, histamine, putrescine, cadaverine, and 2-phenethylamine were not found in the supernatant of KGC1201 culture. Taken together, KGC1201 was confirmed as sufficiently safe for use as a probiotic.

### Probiotic Properties in Intestinal Phase: Bile Salt Resistance and Adhesion to Intestinal Cells

To use LAB as a material for probiotic foods, it is important that a large number of bacteria pass through the digestive tract and settle in the human intestine. Therefore, resistance to bile salt and adhesion ability to intestinal epithelial cells are essential properties for probiotics. Bile salt resistance of KGC1201 measured using bovine bile (0.1% Oxgall) was 98.34%, which was similar to that of KCTC3109 (97.40%) under the same experimental conditions. The evaluation of the adhesion ability to intestinal cells was conducted using colonic epithelial cells (HT-29) under a simulated environment similar to the intestine (Table 2). After incubation with HT-29 cells for 2 h, 90.67% of the initially inoculated KGC1201 remained on the HT-29 cells, which was slightly better than the adhesion rate of KCTC3109 (88.02%). These results suggest that KGC1201 can successfully survive and colonize the human intestine, indicating that KGC1201 has sufficient qualities as a probiotic.

### Probiotic Properties in Gastric Phase: Acid Resistance of KGC1201

**Fig. 1:**
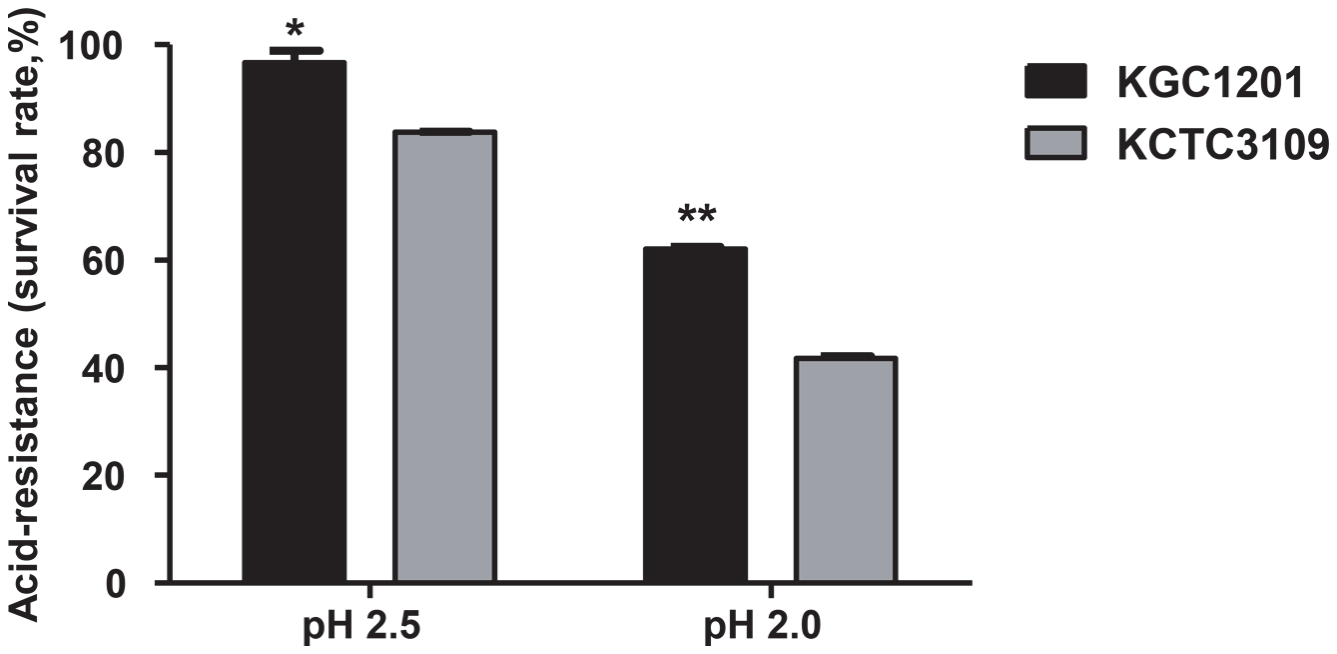
Acid resistance of KGC1201 and KCTC3109. Survival rate (%) = viable cells (log CFU/ml) / initial cells (log CFU/ml)] × 100. The data are presented as mean ± standard error of the mean (*n* = 3). Significant differences compared to *L. casei* type strain KGC3109 were determined using Student’s *t*-tests and indicated as *p < 0.05 and **p < 0.01.

**Fig. 2:**
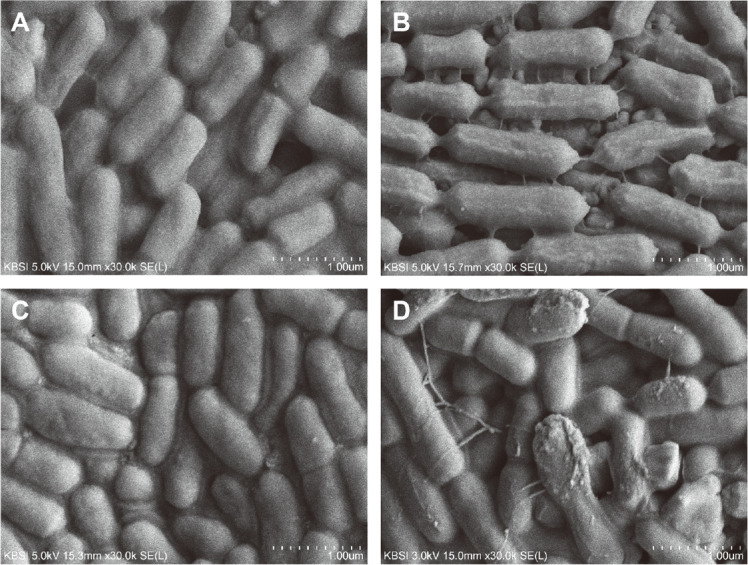
Field emission scanning electron microscopy (FE-SEM) analysis of morphological changes in cells under an acid environment. KGC1201 (**A**) and KCTC3109 (**B**) grown under standard conditions. KGC1201 (**C**) and KCTC3109 (**D**) exposed to acid of pH 2.0 for 3 h.

In the gastric phase, probiotics are exposed to a highly acidic environment that can be detrimental to their survival and function. Resistance to acid stress is therefore one of the most important factors for the survival of LAB, and improved acid resistance has become a crucial criterion when selecting bacteria for industrial use as probiotics. To evaluate the acid resistance of KGC1201, the viability of the cells was monitored in an environment similar to that of the human stomach. After 3 h of exposure to an acidic environment of pH 2.5, the density of KCTC3109 was reduced significantly from 8.26 log CFU/ml to 6.92 log CFU/ml (Fig. 1). On the other hand, the density of KGC1201 decreased slightly from 8.34 log CFU/ml to 8.07 log CFU/ml, showing a survival rate of almost 97%. Under extremely acidic conditions of pH 2.0, the density of KCTC3109 was reduced to 3.44 log CFU/ml, indicating that only 42% of KCTC3109 survived. However, the viable cell density of KGC1201 was 5.17 log CFU/ml under the same conditions, which means that the survival rate of KGC1201 was about 62%, significantly higher than that of KCTC3109. These results were further substantiated using field FE-SEM. After exposure to pH 2.0 for 3 h, KCTC3109 cells were observed to be severely damaged, whereas little morphological change was detected in KGC1201 cells, an observation consistent with the high viability of KGC1201 under acidic conditions (Fig. 2).

### Relative Expression of Acid Resistance Genes

**Fig. 3:**
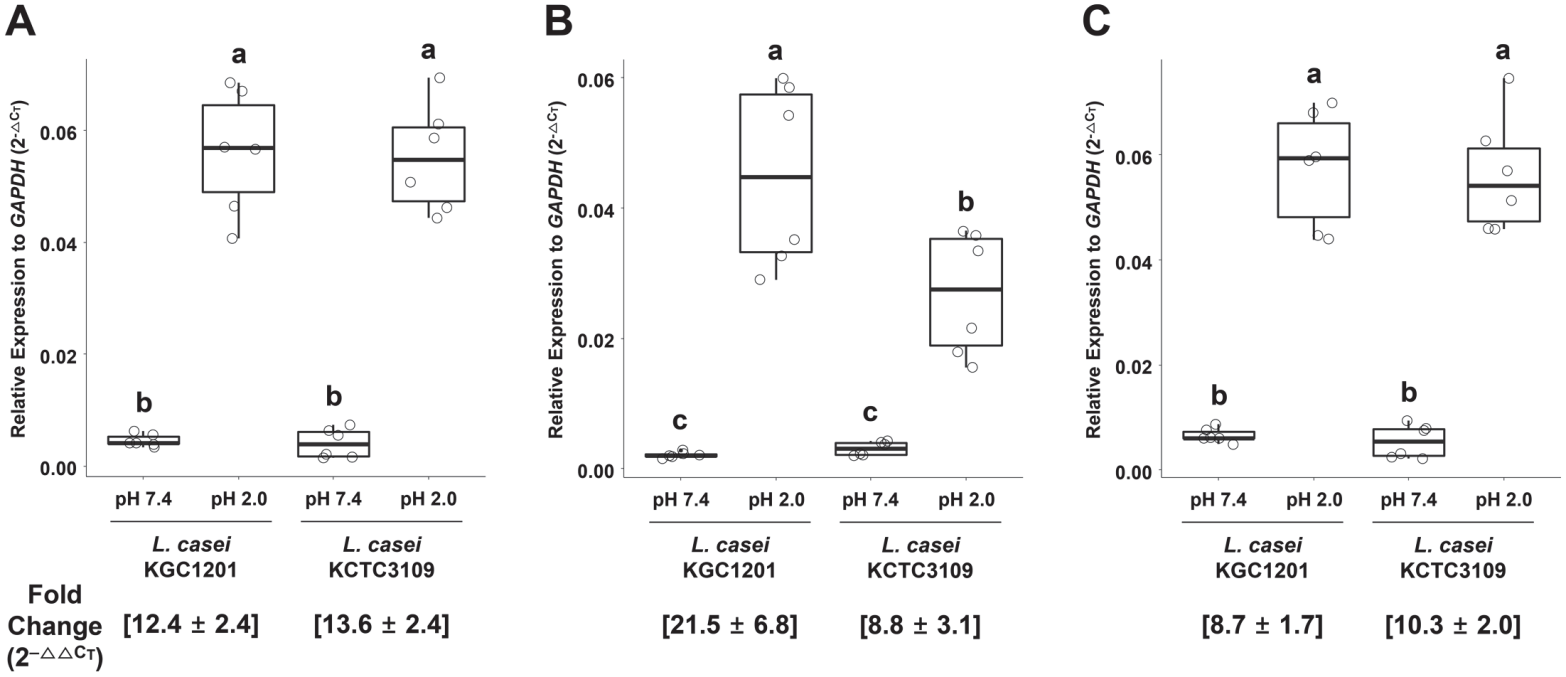
Expression of acid resistance genes relative to GAPDH and fold change in gene expression under acidic conditions (pH 2.0) compared to control conditions (pH 7.4): (A) H^+^/Cl^‒^ exchange transporter, (B) glycosyltransferase, (C) histidine kinase. Expression values were obtained using 2^–ΔC_T_^, and ΔC_T_ values were calculated using the C_T_ acid resistance gene using the [C_T_ acid resistance gene] - [C_T_ *gapdh*]. Fold changes are expressed as mean ± S.D. (*n* = 6) using 2^–ΔΔC_T_^ and ΔΔC_T_ determined by [ΔC_T_ pH 2.0] - [ΔC_T_ pH 7.4]. Horizontal bars, boxes, and whiskers show medians, interquartile ranges, and data ranges, respectively. Different superscript lowercase letters indicate significant differences according to one-way ANOVA and Tukey HSD tests (*p* < 0.05).

The phenotypic trait of higher viability of KGC1201 under acidic conditions may be attributable to the enhanced expression of genes involved in the regulation of intracellular pH, biosynthesis of EPS, or sensing of acidification [40]. Based on genomic analysis of KGC1201, three genes—H^+^/Cl^–^ exchange transporter, glycosyltransferase, and histidine kinase—were selected as acid resistance-related genes. The relative expression levels of these genes were highly elevated in acidic conditions compared to control conditions, by at least 8.7-fold (Fig. 3). However, the fold changes in gene expression of H+/Cl– exchange transporter and histidine kinase were not significantly different between KGC1201 and KCTC3109 (Figs. 3A and 3C). Only glycosyltransferase exhibited a significant change in gene expression, indicating that it was upregulated 21.5-fold in KGC1201, but only 8.8-fold in KCTC3109 (Fig. 3B). Glycosyltransferase plays an important role in the biosynthesis of EPS, and its gene expression is transcriptionally regulated under stressful conditions [41, 42]. Glycosyltransferases in *Mycobacterium marinum* (WcaA) and *Pseudomonas aeruginosa* (WapR) have been characterized as enzymes involved in the biosynthesis of cell wall-associated glycolipids and carbohydrate polymers, creating a natural barrier against environmental stress [43, 44]. Therefore, the higher expression of the glycosyltransferase gene in KGC1201 than in KCTC3109 is likely to increase the survival rate of KGC1201 under acidic conditions through elevated EPS biosynthesis.

### Quantification of EPSs Produced by KGC1201

**Fig. 4:**
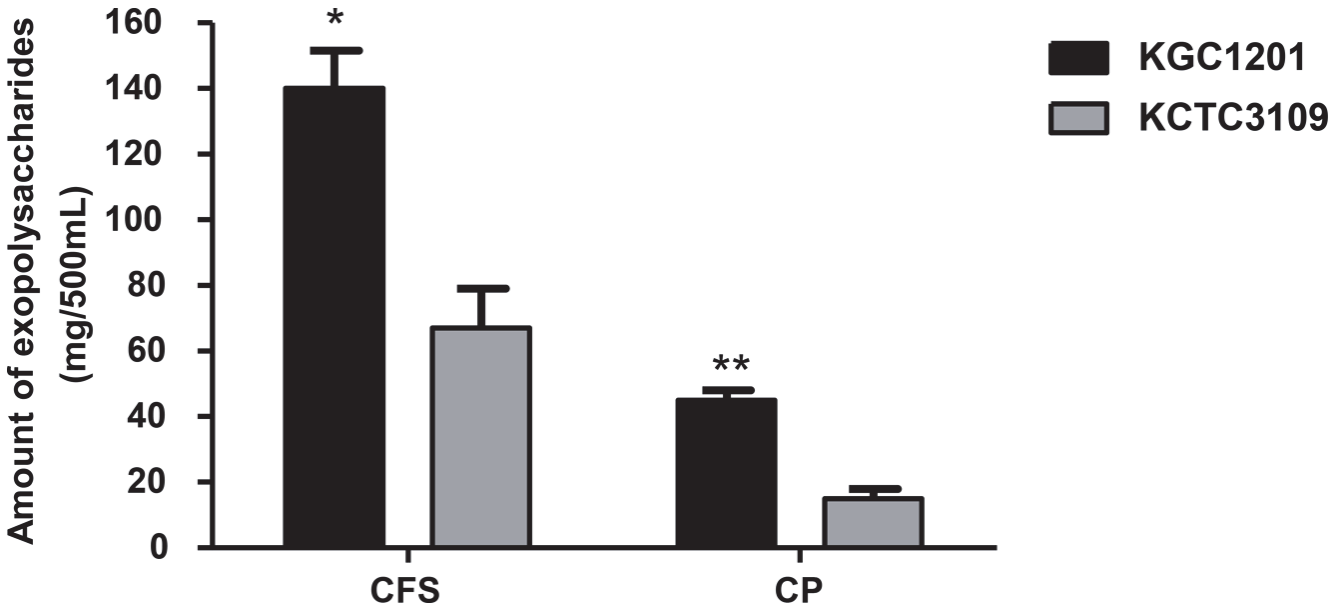
Quantification of exopolysaccharides (EPS). The amount of EPS purified from 500 ml of the culture medium was measured separately as cell-free supernatant (CFS) and cell pellet (CP). The data are presented as mean ± standard error of the mean (*n* = 3). Significant differences compared to KGC3109 were determined using Student’s *t*-tests and indicated as *p < 0.05 and **p < 0.01.

To determine whether EPS biosynthesis was increased in KGC1201, the EPS content isolated from the cell pellets and the cell-free supernatants was quantified, respectively. EPSs isolated from cell pellets include polysaccharides bound to the bacterial cell surface, whereas EPSs from the cell-free supernatants is composed of polysaccharides released into the surrounding medium [27]. As a result, the amount of EPSs contained in 500 ml of the KCTC3109 culture medium was 67 ± 12 mg in the cell-free supernatants and 15 ± 2.9 mg in the cell pellets (Fig. 4). In contrast, KGC1201 produced significantly higher amounts of EPSs than KCTC3109, with 2.1-fold more cell-bound EPSs (140 ± 12 mg) and 3-fold more released EPS (45 ± 2.9 mg). EPS, particularly cell-bound ESP, has been suggested to play a role in protecting cells from environmental stresses such as desiccation, high osmotic pressure, oxidative stress, heat shock, or high acidity [13, 27, 45]. Biofilm constituted by EPSs is the first barrier of the cell and modifying its physicochemical properties, such as membrane mobility and ratio of unsaturated fatty acids, has proved to be an important survival strategy for many microorganisms [11, 12]. Since resistance caused by EPS has been reported to exhibit strain-specific actions in LAB [40], the increased EPS production of KGC1201 may be an intrinsic mechanism attributable to changes in the expression of specific genes such as glycosyltransferase. Indeed, KGC1201 produced a higher amount of EPS than KCTC3109 through modulating the expression of the glycosyltransferase gene, and the increased EPS by this molecular mechanism can be inferred as contributing to the improved acid resistance of KGC1201. In addition, because probiotic EPS confers health benefits such as immune-stimulatory, antitumoral effects and lowering blood cholesterol, as well as being widely used in the food industry as a viscous agent, stabilizer, gelling agent, and emulsifier [27, 40], the high EPS yield of KGC1209 will further increase its utilization value as a probiotic.

Collectively, *L. casei* KGC1201 isolated from ginseng is safe for ingestion as food and has excellent properties as a probiotic. The unique characteristics of the strain were confirmed through 16S rRNA sequencing, whole-genome sequencing, and biochemical properties. In particular, the acid resistance of KGC1201 was superior to that of the *L. casei* type strain KCTC3109. Morphological observation identified less damage to the cell surface of KGC1201, and gene expression studies indicated that the expression level of the glycosyltransferase gene was highly elevated under acidic conditions. EPSs biosynthesized by glycosyltransferase were produced more in KGC1201 than in KCTC3109, which is inferred to better protect KGC1201 cells from strong acidity. Therefore, the superior characteristics of KGC1201 as a probiotic and its acid resistance-related molecular mechanisms may contribute to improving overall human health and well-being.

## Supplemental Materials

Supplementary data for this paper are available on-line only at http://jmb.or.kr.

## Figures and Tables

**Table 1:** Carbohydrate fermentation patterns of *Lacticaseibacillus casei* KGC1201 and *L. casei* type strain KCTC3109.

Carbohydrates	KGC1201	KCTC3109	Carbohydrates	KGC1201	KCTC3109
Glycerol	v	v	Salicin	+	+
Erythritol	-	-	D-cellobiose	+	+
D-arabinose	-	-	D-maltose	+	v
L-arabinose	-	-	D-lactose	+	+
D-ribose	-	-	D-melibiose	+	-
D-xylose	-	-	D-saccharose (sucrose)	v	v
L-xylose	-	-	D-trehalose	+	+
D-adonitol	-	-	Inulin	-	-
Methyl-β-D-Xylopyranoside	-	-	D-melezitose	+	+
D-galactose	+	+	D-raffinose	+	-
D-glucose	+	+	Amidon (starch)	-	-
D-fructose	+	+	Glycogen	-	-
D-mannose	+	+	Xylitol	-	-
L-sorbose	-	-	Gentiobiose	+	+
L-rhamnose	-	-	D-turanose	-	-
Dulcitol	-	-	D-lyxose	-	-
Inositol	-	-	D-tagatose	+	+
D-mannitol	+	+	D-fucose	-	-
D-sorbitol	-	-	L-fucose	-	-
Methyl-α-D-Mannopyranoside	-	-	D-arabitol	-	-
Methyl-α-D-Glucoopyranoside	-	-	L-arabitol	-	-
N-acetylglucosamine	+	+	Potassium gluconate	v	v
Amygdalin	+	+	Potassium 2-ketogluconate	-	-
Arbutin	+	+	Potassium 5-ketogluconate	-	-
Esculin ferric citrate	+	+			

−, not utilized; +, strongly utilized; v, weakly utilized.

**Table 2:** Probiotic characteristics related to bile salt resistance and adhesion ability to intestinal cells of KGC1201 and KCTC3109.

Characteristics		KGC1201	KCTC3109
Bile salt resistance (0.1% Oxgall)	0 h (log CFU/ml)	8.34 ± 0.00	8.26 ± 0.01
	3 h (log CFU/ml)	8.20 ± 0.04	8.04 ± 0.03
	Survival rate (%)	98.34 ± 0.50	97.40 ± 0.41
Adhesion ability to intestinal cells (HT-29)	0 h (log CFU/ml)	7.27 ± 0.01	7.26 ± 0.05
	2 h (log CFU/ml)	6.60 ± 0.02	6.39 ± 0.05
	Adhesion rate (%)	90.67 ± 0.26	88.02 ± 0.63
