# Supplementary material for: Probiotic Characteristics and Safety Assessment of Lacticaseibacillus casei KGC1201 Isolated from Panax ginseng
Source: J Microbiol Biotechnol. 2023 Jan 31;33(4):519–26. doi: 10.4014/jmb.2211.11029 (PMC10164721; doi:10.4014/jmb.2211.11029)
Supplement: Supplementary file 1 [file jmb-33-4-519-supple.pdf]

J. Microbiol. Biotechnol.
https://doi.org/10.4014/jmb.2211.11029

Supplementary Materials

Table S1. Oligonucleotides primers for acid-resistance genes for qRT-PCR analysis of *Lacticaseibacillus casei* KGC1201 and *L. casei* type strain KCTC3190.

**Genes**	**Primers (5'–3')**	**Protein ID**	
		**KGC1201**	**KCTC3109**
H^+/Cl^- exchange transporter	Forward: ATATGGGGCCGTTTGTCTGG<br>Reverse: ACCCAGAAACAAACCGGGAG	P2D_00826	WP_025013040
glycosyl-transferase	Forward: GTCGATATGACGGTGGTCAA<br>Reverse: TCCTCAAGCCAATACCGATAC	P2D_00942	WP_039638894
histidine kinase	Forward: AAAGTCCCCGCATCTCGTTA<br>Reverse: GGCAATGGTCAAATCGCCAC	P2D_01520	WP_052253345
gapdh	Forward: TCGTTTGGCATTCCGTCGTA<br>Reverse: GGGCAGAAATCAGAACACGC	P2D_00822	WP_025013036

Table S2. Endophytic bacteria isolated from *Panax ginseng* root.

Genus	Species	Genus	Species
Lacticaseibacillus	***casei***	*Sporolactobacillus*	*terrae*
Bacillus	*coagulans*		*laevolacticus*
	hisashii	*Clostridium*	*algidixylanolyticum*
	cereus		*beijerinckii*
	wiedmannii		*diolis*
	paranthracis	*Enterococcus*	*durans*
	toyonensis	*Raoultella*	*planticola*
	pacificus		
	proteolyticus		

Table S3. Genome characteristics of KGC1201 and KCTC3109, and average nucleotide identity (ANI) between their sequences.

**Strain**	**KGC1201**	**KCTC3109**
Total length (bp)	2,751,907	2,952,961
GC content (%)	47.73	47.88
ANI value (%)	99.92	

Table S4. Minimum inhibitory concentrations (MIC) of KGC1201 for 8 antibiotics.

**Antibiotics**[a]	**AMP**	**GEN**	**KAN**	**STR**	**ERY**	**CLI**	**TET**	**CHL**
Cut-off value (mg/L)[b]	4	32	64	64	1	1	4	4
Observed MICs	0.094	0.094	> 256	64–96	0.25	0.50	0.38	3
Assessment	S	S	R	R	S	S	S	S

[a]AMP, ampicillin; GEN, gentamycin; KAN, kanamycin; STR, streptomycin; ERY, erythromycin; CLI, clindamycin; TET, tetracycline; CHL, chloramphenicol.

[b]Values were determined according to European Food Safety Authority (EFSA) guidelines, 2018.

[c]S, susceptible; R, resistant.

Table S5. Enzymatic activities of KGC1201.

Enzymes	Activity	Enzymes	Activity
Alkaline phosphatase	+	Naphthol-AS-BI-phosphohydrolase	+
Estrase	+++	α-galactosidase	–
Esterase lipase	+	β-galactosidase	+++
Lipase	–	β-glucuronidase	–
Leucine arylamidase	+++	α-glucosidase	–
Valine arylamidase	+++	β-glucosidase	+++
Crystinearylamidase	+	N-acetyl-β-glucosaminidase	–
Trypsin	–	α-mannosidase	–
α-chymotrypsin	–	α-fucosidase	–
Acid phosphatase	+++		

–, no activity; +, poor activity; ++, moderate activity; +++, strong activity.

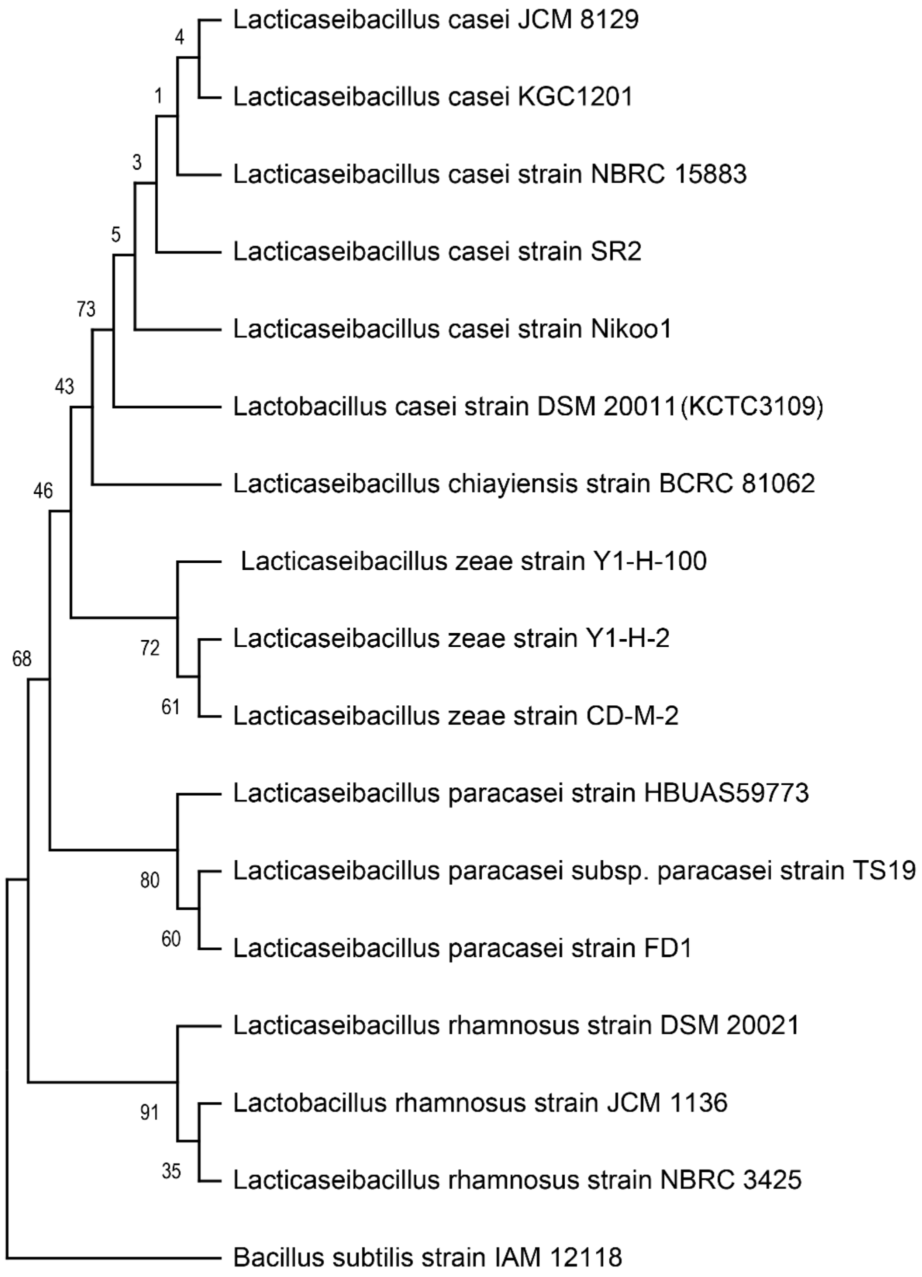

Figure S1. 16S rRNA phylogenetic tree based on the sequence data obtained from the NCBI database. The relationships between KGC1201 and other strains are shown. The tree was constructed using the Maximum Likelihood method and Kimura 2-parameter model. In the bootstrap test (500 replicates), the proportion of replication trees clustered with the associated taxonomies is displayed next to the branch. *Bacillus subtilis* was used as an outgroup strain for tree rooting. Evolutionary analyses were conducted in MEGA11.

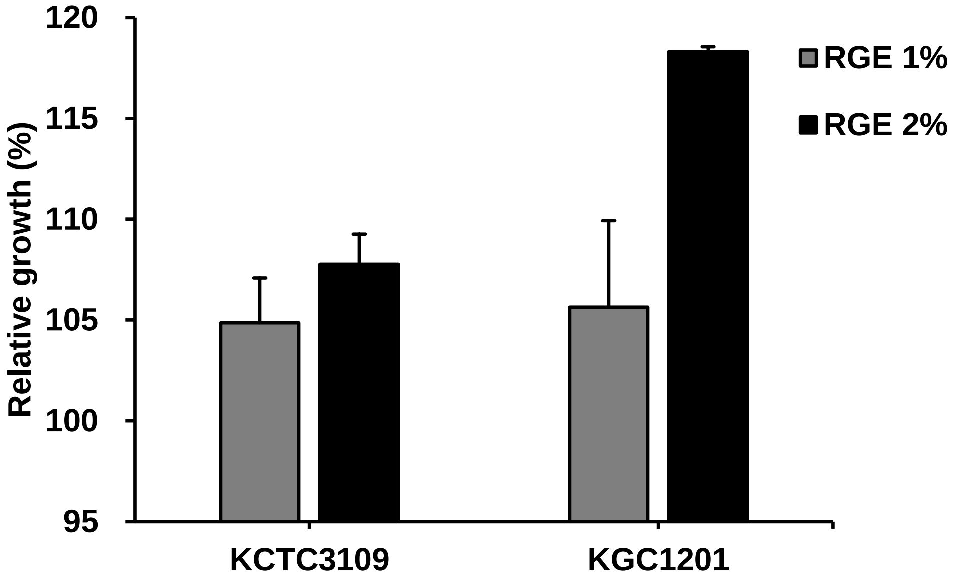

Figure S2. Relative cell growth of KGC1201 and KCTC3109 dependent on the concentration of red ginseng extracts (RGE). Cells grown in MRS broth containing either 0%, 1% and 2% RGE were counted on agar plates, and relative growth was determined in proportion to the number of cells in 0% RGE medium. The data are presented as mean ± standard error of the mean (*n* = 2).

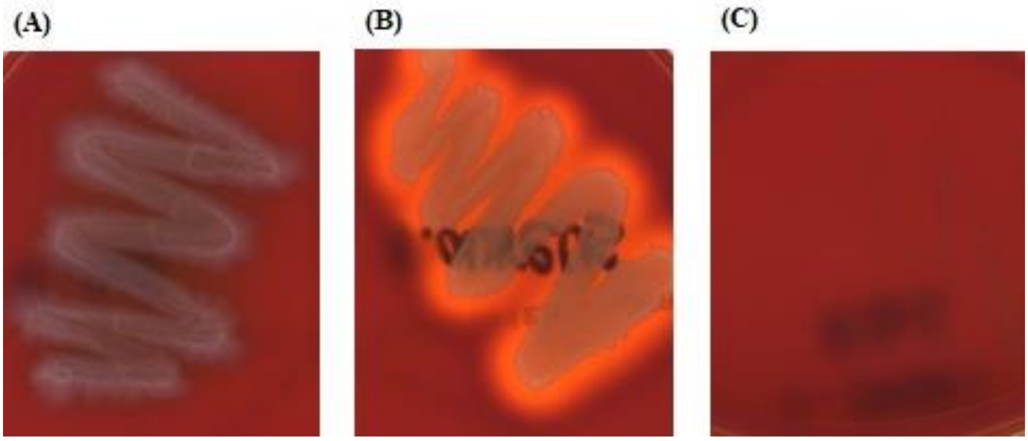

Figure S3. Hemolytic activity of KGC1201, *Escherichia coli*, and *Staphylococcus aureus*. *E. coli* KCTC2441was used as a positive control for α-hemolysis (A) and *S. aureus* NCTC10788 was used as a positive control of β-hemolysis (B). The hemolytic properties of KGC1201 (C) and controls were assessed by forming clear zones around the colonies on sheep blood agar plates.
